# A Population-Based Study of Diabetes during Pregnancy in Spain (2009–2015): Trends in Incidence, Obstetric Interventions, and Pregnancy Outcomes

**DOI:** 10.3390/jcm9020582

**Published:** 2020-02-21

**Authors:** Ana López-de-Andrés, Napoleón Perez-Farinos, Valentín Hernández-Barrera, María A. Palomar-Gallego, David Carabantes-Alarcón, José J. Zamorano-León, Javier De Miguel-Diez, Rodrigo Jimenez-Garcia

**Affiliations:** 1 Preventive Medicine and Public Health Teaching and Research Unit, Health Sciences Faculty, Rey Juan Carlos University, Alcorcón, 28922 Madrid, Spain; ana.lopez@urjc.es (A.L.-d.-A.); valentin.hernandez@urjc.es (V.H.-B.); 2 Public Health and Psychiatry Department, Faculty of Medicine, Universidad de Malaga, 29071 Malaga, Spain; 3 Basic Science Department, Health Sciences Faculty, Rey Juan Carlos University, Alcorcón, 28922 Madrid, Spain; mariaangustias.palomar@urjc.es; 4 Department of Public Health & Maternal and Child Health, Faculty of Medicine, Universidad Complutense de Madrid, 28040 Madrid, Spain; dcaraban@ucm.es (D.C.-A.); jjzamorano@ucm.es (J.J.Z.-L.); rodrijim@ucm.es (R.J.-G.); 5 Respiratory Department, Hospital General Universitario Gregorio Marañón, Facultad de Medicina, Universidad Complutense de Madrid, Instituto de Investigación Sanitaria Gregorio Marañón (IiSGM), 28009 Madrid, Spain; javier.miguel@salud.madrid.org

**Keywords:** gestational diabetes mellitus, type 1 diabetes mellitus, type 2 diabetes mellitus, pregnancy, hospitalizations, outcomes

## Abstract

(1) Background: We examined trends in incidence and outcomes in women with existing type 1 diabetes mellitus (T1DM), type 2 diabetes mellitus (T2DM) and gestational diabetes mellitus (GDM) compared with a control group without diabetes. (2) Methods: This was an observational, retrospective epidemiological study using the National Hospital Discharge Database. (3) Results: There were 2,481,479 deliveries in Spain between 2009 and 2015 (5561 mothers with T1DM, 4391 with T2DM, and 130,980 with GDM). Incidence and maternal age of existing diabetes and GDM increased over time. Women with T2DM were more likely to have obstetric comorbidity (70.12%) than those with GDM (60.28%), T1DM (59.45%), and no diabetes (41.82%). Previous cesarean delivery, preeclampsia, smoking, hypertension, and obesity were the most prevalent risk factors in all types of diabetes. Women with T1DM had the highest rate of cesarean delivery (Risk Ratio (RR) 2.34; 95% Confidence Interval (CI) 2.26–2.43) and prolonged maternal length of stay. Labor induction was higher in T2DM (RR 1.99; 95% CI 1.89–2.10). Women with T1DM had more severe maternal morbidity (RR 1.97; 95% CI 1.70–2.29) and neonatal morbidity (preterm birth, RR 3.32; 95% CI 3.14–3.51, and fetal overgrowth, RR 8.05; 95% CI 7.41–8.75). (4) Conclusions: existing and GDM incidence has increased over time. We found differences in the prevalence of comorbidities, obstetric risk factors, and the rate of adverse obstetric outcomes among women with different types of diabetes. Pregnant women with diabetes have the highest risk of adverse pregnancy outcomes.

## 1. Introduction

Diabetes mellitus during pregnancy is associated with an increased risk for severe maternal and neonatal morbidity [1,2]. Type 1 diabetes mellitus (T1DM) or type 2 diabetes mellitus (T2DM) before pregnancy (existing diabetes) is associated with an around four times higher risk of preeclampsia and hypertension and almost twice the likelihood of cesarean delivery [3]. Gestational diabetes is associated with a greater risk of T2DM in the future [4] and greater neonatal morbidity when compared with pregnant women without diabetes (Odds Ratio (OR) 1.16; 95% Confidence Interval (CI) 1.04–1.30) [1]. Recently, Tinker et al. [5] reported strong associations between the presence of diabetes in pregnant women and several types of birth defects and fetal overgrowth [6].

The prevalence of different types of diabetes among pregnant women is rising worldwide [7,8,9,10,11]. Population-based studies in Spain showed that the number of deliveries in women with existing diabetes and gestational diabetes has increased between 2001 and 2008 [3,12]. In the United Kingdom, a study also reported an increment in deliveries of women with existing diabetes between 1995 and 2012, and higher rates of adverse pregnancy outcomes and obstetric interventions [13]. Similar findings have been reported in Canada and Sweden [14,15]. However, in the United States a recent study concluded that from 2012 to 2016 the prevalence of gestational diabetes rose from 5.2% to 5.6%, and that the prevalence of existing diabetes remained unchanged at 0.8% [16].

It has been reported that, as the maternal age increases and the prevalence of obesity continues to rise, more women will be affected by the complications associated with existing diabetes and gestational diabetes [11].

The aim of the present study was to examine nationwide trends in Spain, from the years 2009 to 2015, in the incidence of deliveries, obstetric interventions, and obstetric outcomes among women with T1DM, T2DM, and gestational diabetes, and to compare these groups with women without diabetes.

## 2. Materials and Methods

### 2.1. Design, Setting, and Participants

This observational retrospective epidemiological study was conducted using the Spanish National Hospital Discharge Database (SNHDD) from 1 January 2009 to 31 December 2015. The SNHDD provides de-identified detailed medical information on over 95% of admissions to Spanish public and private hospitals, including discharge diagnoses (up to 14) and procedures (up to 20) performed during the hospital stay using the International Classification of Disease, 9th Revision, Clinical Modification (ICD-9-CM) codes. Details of the SNHDD have been described in previous studies by our group [3,12].

For the study purpose, we identified delivery admissions using the ICD-9-CM procedure and diagnosis codes, a methodology defined and validated by Kuklina et al. [17].

ICD-9-CM codes were used to classify women with diabetes: T1DM (250.1×, 250.3×), T2DM (250.0×, 250.2×), and gestational diabetes (648.8×), with no other codes for existing diabetes. All other deliveries without any of the previous ICD-9-CM codes were included in the “Without diabetes” group.

In Spain, official screening recommendations for GDM have remained unchanged in recent decades, as described by the Spanish Group of Diabetes and Pregnancy and the Spanish Ministry of Health [18,19]. Recommended universal screening for GDM in Spain is conducted using a two-step approach that includes a screening 50-g glucose challenge test at weeks 24–28 of gestation followed by a 100-g oral glucose tolerance test if the glucose challenge test is positive (≥140 mg/dL). Diagnosis is confirmed by any two plasma glucose level values at or above 105 mg/dL, 190 mg/dL, 165 mg/dL, and 145 mg/dL at fasting, 1 h, 2 h, and 3 h, respectively. For women with a high risk of GDM (>35 years of age, obesity, personal history of GDM, family history of type 2 diabetes in first- or second degree relatives, high-risk ethnicity), screening for GDM is recommended during the first trimester of pregnancy using the same approach [18,19].

### 2.2. Main Outcomes Measures

To identify comorbidity and obstetric risk factors among women with diabetes we used the Obstetric Comorbidity Index (OCI) [20]. The OCI is a weighted algorithm that assigns points for the presence of preexisting comorbidities, substance-related conditions, pregnancy-related conditions, and advanced maternal age (≥35 years). Batteman et al. in 2013 used a cohort of 569,882 women as a development sample and 284,941 women as a validation sample to define the OCI. Using the development sample, they constructed a multivariable logistic regression model using a fully stepwise selection algorithm that requires covariates to have a *p*-value ≤ 0.05 for both entry and retention in the model. The dependent variable was the presence of maternal end-organ injury or death during the delivery hospitalization through 30 days postpartum. The candidate-independent variables included the 24 maternal comorbidities defined as well as maternal age categorized as <19, 20–34, 35–39, 40–44, and >44 years at the time of last menstrual period. The final model included several maternal conditions and maternal age. Using the results from the final logistic regression model, those conditions with a beta coefficient ≤0.15 are assigned a weight of zero, and for each 0.3 increase in the beta coefficient, the weight assigned to individual conditions is increased by 1 point. Patient comorbidity index is then obtained by summing the weights for all comorbidities present and adding it to the relevant weight for the patient age. The performance characteristics of the newly derived score were then assessed using the validation cohort. The discrimination of the model was evaluated by calculating the area under the receiver operating curve (cstatistic) [20]. The ICD-9 codes used to identify the conditions of the OCI are shown in Supplementary Table S1.

Among the obstetric interventions, labor induction (ICD-9-CM codes: 73.4, 73.01, 73.1), cesarean delivery (ICD-9-CM codes: 74.0-74.4, 74.99), forceps/vacuum extraction (ICD-9-CM codes: 72.0, 72.1, 72.21, 72.29, 72.31, 72.39, 72.4, 72.6; 72.51, 72.52, 72.53, 72.54; 72.71, 72.79), and episiotomy (ICD-9-codes: 73.6) were analyzed.

Severe maternal morbidity was defined as the presence of at least one of the 21 indicators of maternal morbidity as specified by the Centers for Disease Control and Prevention using the ICD-9-CM [21].

We considered prolonged maternal length of stay as length of hospital stay over 4 days following cesarean delivery and over 2 days following vaginal birth as described by Metcalfe et al. [14]. Using ICD-9-CM codes we defined neonatal complications as preterm birth (644.2×) and fetal overgrowth (656.6). We could not analyze the presence of macrosomia in the infant because we identified women for delivery admissions and according to the SNHDD methodology, “macrosomia” (ICD-9MC code 766.0; oversize fetus weight of 4500 grams or more) can only be included as a diagnosis in the child´s discharge report but not in the mother’s report [3,12].

Maternal in-hospital mortality was defined by the proportion of women who died during delivery admission for each year of study.

### 2.3. Statistical Methods

The incidence rates of delivery admissions in women with T1DM, T2DM, and gestational diabetes per 10,000 deliveries were estimated using the methods described in our previous studies [3,12].

Descriptive statistical analysis included proportions for categorical and means with standard deviations for continuous variables. For bivariable analysis, we used the χ^2^ test, *t* student test, and ANOVA, as required.

To examine temporal trends in the prevalence of comorbidities and obstetric interventions we used non parametric test for trends. We used the χ^2^ test to examine the association of diabetes types with neonatal morbidity and severe maternal outcomes.

We estimated relative risk of obstetrical interventions and severe maternal and neonatal complications (preterm birth and fetal overgrowth) between diabetes types using multivariable log binomial models. This method has been previously used by Metcalfe et al. and details on how to conduct log binomial models with STATA are described elsewhere [14,22]. These models were adjusted by age and OCI and women without diabetes were used as reference category. We consider that the OCI is better than common confounders to adjust multivariable models because it is an important tool for summarizing comorbidity and for confounding control and has been previously validated and used by other authors [14,20,23,24,25].

We performed all analysis with Stata version 10.1 (Stata, College Station, TX, USA). Statistical significance was set at *p* < 0.05 (2-tailed).

### 2.4. Ethical Aspects

Retrospective use of de-identified register data does not require ethical approval or informed consent according to Spanish legislation. The Spanish Ministry of Health (SMH) provided the database and gave us permission to use the data after we signed an engagement in which we legally committed to: (1) under no circumstances export the entire database or make partial exports that could allow the generation of the same through aggregation or identification of natural persons or reporting units and, (2) destroy the file or data provided and all the copies made of it once the period of time for which the data was required had elapsed.

## 3. Results

Overall, there were 2,481,479 deliveries in Spain between 2009 and 2015, 9952 coded with existing diabetes (55.87% with T1DM and 44.13% with T2DM), and 130,980 with gestational diabetes.

### 3.1. Incidence of Deliveries, Obstetric Interventions and Obstetric Outcomes among Women with Existing and Gestational Diabetes

#### 3.1.1. Time Trends in the Incidence and Demographic Characteristics of Deliveries in Women with Existing and Gestational Diabetes

The incidence rate per 10,000 deliveries increased significantly over time in all types of diabetes (*p* < 0.001), as can be seen in Table 1. The incidence of T1DM in pregnancy rose from 17.98 per 10,000 deliveries in 2009 to 23.9 in 2015 (*p* < 0.001) and for T2DM increased from 14.56 in 2009 to 22.4 per 10,000 deliveries in 2015 (*p* < 0.001), representing an increase >60% over the study period. Gestational diabetes in pregnancy also increased from 445.24 to 549.37 per 10,000 deliveries over the study period (*p* < 0.001).

Mean maternal age increased over time in pregnant women with T1DM and gestational diabetes (all *p* < 0.001) and was stable around 34 years old in those with T2DM.

#### 3.1.2. Comorbidities and Obstetrical Risk Factors of Deliveries in Women with Existing and Gestational Diabetes

As can be seen in Table 2, over the entire period obstetric comorbidities were more common among pregnant women with diabetes than those without, with 70.12% of women with T2DM, 60.28% of women with gestational diabetes, and 59.45% of women with type1 diabetes having at least one condition included in the OCI, with an equivalent figure for non-diabetic women of 41.82%.

Previous cesarean delivery was the most prevalent obstetrical risk factor in all types of diabetes, with proportions of around 18% for those with T1DM or T2DM, 11% among those with gestational diabetes, and 8% for women without diabetes. Besides this risk factor, the most common risk factors for each type of diabetes were as follows: in women with T1DM, 6.28% had mild preeclampsia and 6.01% were tobacco users; in women with T2DM, 10.79% were obese and 10.48% had hypertension; and in women with gestational diabetes, 5.7% were tobacco users and 5.26% were obese (Table 2).

The types of comorbidities present differed between types of diabetes. In pregnant women with T2DM, prevalence of hypertension and obesity was fivefold and twofold higher, respectively, compared with women with gestational diabetes. Prevalence of mild and severe preeclampsia was higher in women with T1DM than in those with gestational diabetes (6.28% and 2.21% vs. 1.86% and 0.65%, respectively) (Table 2).

**Figure 1:**
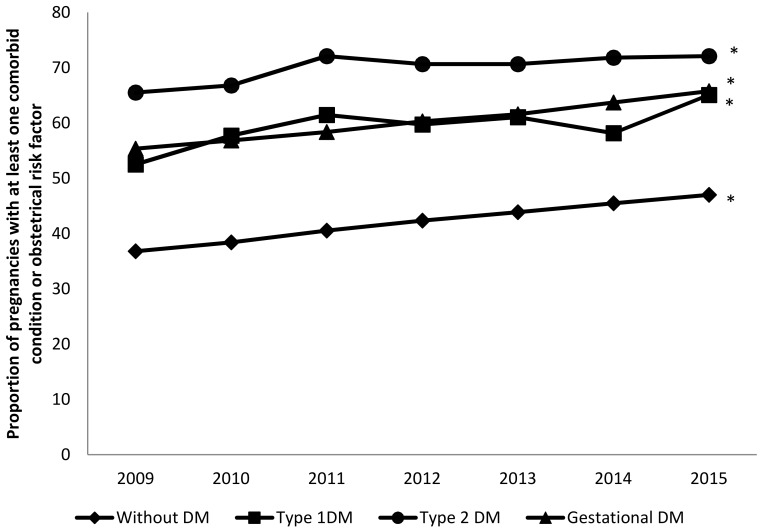
Temporal trends in the proportion of pregnancies in women with type 1 diabetes (T1DM), T2DM, and gestational diabetes with at least one comorbid condition (excluding diabetes) compared with women without diabetes. * *p* < 0.05 for trends.

OCI increased significantly over time (in T2DM: from 65.5% in 2009 to 72.09% in 2015; in T1DM: from 52.48% in 2009 to 65.05% in 2015; and in gestational diabetes: from 55.33% in 2009 to 65.77% in 2015; all *p* < 0.001). Prevalence of comorbidity according to the OCI was higher in pregnant women with T2DM than in women with other types of diabetes in all the study years, as can be seen in Figure 1.

#### 3.1.3. Impact of Diabetes Mellitus Types on Obstetrical Interventions and Maternal and Neonatal Health Outcomes

Shown in Table 3 is the impact of diabetes types on obstetrical interventions and maternal and neonatal health outcomes. Labor induction was highest in women with T2DM (30.4%), followed by T1DM (29.58%) and gestational diabetes (22.55%). After controlling for age and OCI using multivariable models, and using women without diabetes as the reference, the probability of labor induction was around twice higher for T1DM and T2DM and 1.45 for gestational diabetes. The use of labor induction increased significantly over time for pregnant women with all types of diabetes (*p* < 0.001) as can be seen in Figure S1.

Pregnant women with T1DM had the highest rate of cesarean delivery (56.86%), followed by women with T2DM (47.46%) and women with gestational diabetes (28.88%), as can be seen in Table 3. After multivariable adjustment the probability of undergoing a cesarean delivery was 2.34, 1.83 and 1.18 times higher for T1DM, T2DM, and gestational diabetes, respectively, compared to non-diabetic women. However, as can be seen in Figure S2, the rate of cesarean delivery remained constant among women with T1DM (*p* = 0.860) and gestational diabetes (*p* = 0.493) but decreased from 50.79% to 40.92% in women with T2DM (*p* = 0.008).

The use of forceps/vacuum extraction was similar in those with and without diabetes, whereas episiotomy was conducted in a significantly lower proportion of women with any type of diabetes than those without the disease (Table 3).

Pregnant women with T1DM had more severe maternal morbidity and neonatal morbidity (preterm birth and fetal overgrowth), followed by women with T2DM, women with gestational diabetes, and women without diabetes (Table 3).

The adjusted risk of suffering severe maternal morbidity, compared with women without diabetes, was significantly higher for T2DM (RR 1.97; 95% CI 1.70–2.29) and for T2DM (RR 1.25; 95% CI 1.02–1.54). After adjusting by OCI and age, gestational diabetes did not increase the risk of severe maternal morbidity.

Women with T2DM had 3.32 higher probabilities of a preterm birth and 8.05 of fetal overgrowth when compared with women without diabetes. Equivalent figures for T1DM were 2.15 and 5.55, and for gestational diabetes, 1.18 and 2.63, respectively.

No significant changes overtime was observed in the rate of severe maternal morbidity in women with diabetes (Figure S3). The rate of preterm births decreased significantly among pregnant women with T2DM and gestational diabetes from 16.11% and 8.13% in 2009 to 12.47% and 7.43%, *p* = 0.05, respectively (Figure S4). No temporal changes in preterm birth rate were observed for women with T1DM (*p* = 0.121) (Figure S4). The rate of fetal overgrowth increased significantly in pregnant women with gestational diabetes over time (3.17% in 2009 vs. 3.64% in 2015; *p* = 0.007). No changes in fetal overgrowth were observed for women with T1DM (*p* = 0.540) or women with T2DM (*p* = 0.456) from 2009 to 2015 (Figure S5).

Regarding and prolonged maternal length of stay the risk was 3.09 (95% CI 2.95–3.23) times higher among T1DM women and 2.22 (95% CI 2.09–2.35) times for those suffering T2DM compared with non-diabetic women. However, the proportion of prolonged maternal length of stay was very similar in case of gestational diabetes (11.19% vs. 10.49%; RR 1.02; 95% CI 0.99–1.03).

Maternal in-hospital mortality was very low in all groups of pregnant women (0% in women with T1DM; 0.05% in women with T2DM; 0.01% in women with gestational diabetes; and 0.01% in the control group).

## 4. Discussion

This population-based study showed that incidence of both existing diabetes and gestational diabetes in pregnancy increased in Spain over the period 2009–2015. As we expected, gestational diabetes had the highest incidence rate. Unlike our earlier findings [12], the increasing number of pregnancies in women with gestational diabetes observed in our study is consistent with studies conducted in other countries [7]. In the United States, the prevalence of gestational diabetes increased from 3.7% in 2000 to 5.8% in 2010 and existing diabetes only from 0.7% to 0.9% [8]. In Scotland, across a 15-year period (1998–2013) the number of pregnancies with T1DM increased significantly by 44% and for T2DM by 90% [10]. A population-based study in Spain showed that the incidence of deliveries of women with existing diabetes increased from 20 to 27 per 10,000 deliveries between 2001 and 2008 [3]. Observed increments in the prevalence of existing and gestational diabetes might be explained by increasing maternal age and obesity prevalence [11,15].

Our study highlights key differences in comorbidity and risk factors, in obstetric management and pregnancy outcomes by diabetes type. Outcomes in women with T1DM and T2DM are worse than in women with gestational diabetes. However, when compared with women without diabetes, the increment of adverse outcomes in women with gestational diabetes is remarkable. Metcalfe et al. [14] concluded that different management strategies for each type of diabetes may be needed to modify the risk of adverse perinatal and obstetric outcomes.

Obesity was one of the most prevalent comorbid conditions in pregnant women with T2DM and gestational diabetes. Timur et al. [26] found that the combination of obesity and diabetes increased cesarean delivery rates, the number of macrosomic neonates, and neonatal intensive care unit admission rates.

Smoking in pregnancy is a well-known risk factor for adverse fetal and maternal outcomes [27]. In our study tobacco smoking was a risk factor found in women with all types of diabetes analyzed. An Italian population-based study described a significant interaction between tobacco smoking and existing diabetes, which increased the risk of preterm birth by 11.7% and congenital anomalies by 2.2% [28].

As we expect, preeclampsia and hypertension were frequent in pregnant women with existing diabetes [3], which is known to impact neonatal outcomes [1], such as preterm birth. The incidence of early preterm birth is considerably increased in women with T1DM and microalbuminuria, which is mostly attributable to early development of preeclampsia [29].

We agree with other authors’ findings that in diabetic women, deliveries were more likely to require labor induction or cesarean than among non-diabetic women after multivariable adjustment [1,2,3,12]. Stogianni et al. [2] identified excessive weight gain during the pregnancy and/or obesity, as well as advanced maternal age as risk factors for higher odds of cesarean section among women with diabetes.

The lower number of episiotomies among T1DM than T2DM patients reflects increased incidence of pre-term deliveries and increased incidence of cesarean sections. As can be seen in Table 3, cesarean sections are found in 56.86% of T1DM versus 47.46% of T2DM women and equivalent figures for pre-term deliveries are 21.96% and 15.03%, respectively.

Severe maternal and neonatal morbidity were significantly higher among women with existing diabetes followed by women with gestational diabetes. A large US cohort concluded that neonates born to mothers with existing diabetes had 2.27 (95% CI 1.95−2.64) times higher risk of composite severe neonatal morbidity compared to non-diabetic mothers, and 1.96 (95% CI 1.63−2.35) times higher risk than women suffering gestational diabetes [1].

We agree with other studies’ findings that the proportion of deliveries with fetal overgrowth among women with diabetes was higher than among those with by gestational diabetes [30]. Furthermore, we found that fetal overgrowth was codified more frequently among T1DM than in T2DM pregnancies. Several previous investigations obtained the same results [6,30]. Possible reasons have been suggested for this finding, including a decreasing rate of microangiopathy among T1DM women, improvement in care that result in lower preterm delivery, or interactions with other determinants of birth weight, such as pre-pregnancy body mass index (BMI) [6,30,31,32]. It has been described that women with T1DM generally gain more weight during pregnancy. T1DM in combination with overweight or obesity constitutes a higher risk for large for gestational age infant than either condition alone [32]. Ladfors et al. [6] concluded that besides diabetes type, gestational weight gain was a major risk factor for fetal overgrowth, and glycemic control was a risk factor only in women with T1DM.

We found that women with T2DM had higher obstetric morbidity than women with T1DM, which may reflect a higher prevalence of obesity, advancing maternal age, and modest increases in the size of ethnic at-risk populations, as described by other studies [10]. Unfortunately, in the present study, data on maternal BMI and ethnicity were unavailable in the database and therefore the effect of increased BMI and high-risk ethnicity could not be assessed. In our population, we may hypothesize that the most significant factor was the increase in overweight/obesity. In this respect, there is strong evidence that the prevalence of overweight/obesity among diabetic women of childbearing age significantly rose during the study period in Spain [33].

The main strength of this study was the use of a large population-based database of 2,481,479 deliveries that reflected trends over a 7-year period and permitted the stratification of outcomes by diabetes type. This database undergoes periodic audits that warranty the validity and completeness of the data. However, this study was subject to several limitations. As this study is based on hospital administrative data, we cannot identify unscreened pregnancies. It is possible that some Spanish regions or hospitals may have used the Hyperglycemia and Adverse Pregnancy Outcome Study/International Association of Diabetes and Pregnancy Study Groups (HAPO/IADPSG) criteria to define GDM, even through the Spanish medical societies and Health Authorities have not changed the recommendations over the study period [18,19]. Other authors have suggested that changes over time in the diagnostic criteria may partly explain the temporal increase in gestational diabetes [1,7].

Clinical data regarding diabetes control and treatments, as well as indications for obstetric intervention are not collected by the SNHDD.

Previous validation studies done with hospital discharge databases have shown that this data source is useful to accurately identify women with gestational diabetes and existing diabetes [34,35,36,37]. Existing diabetes coding validity was found to have a sensitivity of 99.3% and a positive predictive value of 46.4% [34]. However, other validation studies found very high positive predictive value over 95%, these differences are based on the choice of the gold standard [35,36]. Gestational diabetes coding in outpatient and inpatient Canadian databases combined was highly sensitive (92%) and specific (97%), and the authors of the study concluded that can be used to estimate the burden of disease at the population level [37].

The prevalence of smoking found in our investigation was very low. A previous study has found that tobacco use is underreported in discharge diagnoses in hospital databases [38]. In our opinion the main reason for this is that according to the SNHDD methodology the primary/main diagnosis is defined as the condition which, after proper investigation, is considered the reason why the patient was admitted to the hospital. The secondary diagnosis includes those diseases or risk factors that coexist with the primary diagnosis at the time of admission or were detected during the hospitalization and that, in the opinion of the treating physician, may have affected the patient’s progress or treatment plan. Other possible reasons include that those who codify may not record risk factors owing to time constraints when performing data abstraction or that when time for coding is limited, coders tend to include more severe conditions but not risk factors.

In our sample that prevalence of obesity was higher in women with T2DM than in those with GDM. Unfortunately, in the present study, data on maternal BMI were unavailable in the database and therefore the precise effect of increased BMI could not be assessed. The existence of a codification bias, with obesity being codified more frequently among diabetic than non-diabetic women with the same BMI can, therefore, not be assessed and discharged. However, as commented before the prevalence of overweight/obesity in among T2DM Spanish women with of childbearing age increased during the study period [33].

Finally, results and *p* values may reflect the large numbers of patients involved rather than clinical differences.

## 5. Conclusions

We conclude that existing diabetes and gestational diabetes incidence have increased significantly over time, and we found differences in the prevalence of comorbidities, obstetric risk factors, and the rate of adverse obstetric outcomes among women with different types of diabetes.

To improve pregnancy outcomes in women with existing diabetes and gestational diabetes, better knowledge of the obstetric risk associated with different types of diabetes is necessary.

## Figures and Tables

**Table 1:** Incidence rate and demographic characteristics of delivery admissions in Spain, 2009–2015, according to diabetes mellitus (DM) status.

		2009	2010	2011	2012	2013	2014	2015	*p*
Without DM	N	373,235	357,622	347,589	333,999	310,116	308,120	309,866	
	Rate per 10,000 deliveries	9522.2	9455.37	9443.42	9427.25	9384.65	9364.95	9405.12	<0.001
	Maternal age, mean (SD)	30.5 (5.56)	30.68 (5.55)	31 (5.51)	31.17 (5.54)	31.35 (5.56)	31.49 (5.57)	31.67 (5.6)	<0.001
Type 1 DM	N	705	771	789	859	811	865	761	
	Rate per 10,000 deliveries	17.98	20.38	21.43	24.24	24.54	26.29	23.09	<0.001
	Maternal age, mean (SD)	31.52 (5.03)	31.7 (5.24)	31.89 (5.19)	31.81 (5.43)	32.15 (5.13)	32.32 (5.06)	32.53 (5.2)	0.001
Type 2 DM	N	571	545	591	586	668	692	738	
	Rate per 10,000 deliveries	14.56	14.41	16.05	16.54	20.21	21.03	22.4	<0.001
	Maternal age, mean (SD)	33.58 (5.78)	33.9 (5.52)	34.26 (5.28)	34.38 (5.19)	34.21 (5.62)	34.22 (5.26)	34.47 (5.3)	0.073
Gestational DM	N	17,452	19,283	19,106	18,847	18,855	19,337	18,100	
	Rate per 10,000 deliveries	445.24	509.83	519.07	531.96	570.58	587.72	549.37	<0.001
	Maternal age, mean (SD)	33.19 (5.07)	33.29 (5.01)	33.46 (4.96)	33.67 (4.99)	33.88 (5.02)	34.06 (4.95)	34.16 (5.04)	<0.001

*p* < 0.05 for time trend.

**Table 2:** Types of preexisting comorbidities and obstetrical risk factors in the current pregnancy as defined by the Obstetrical Comorbidity Index, according to diabetes mellitus (DM) status.

	Without DM (*n* = 2,340,547)	Type 1 DM (*n* = 5561)	Type 2 DM (*n* = 4391)	Gestational DM (*n* = 130,980)	*p*
Maternal age, mean (SD)	31.1 (5.57)	32 (5.19)	34.17 (5.42)	33.67 (5.02)	<0.001
15–24 years, *n* (%)	313,139 (13.38)	489 (8.79)	174 (3.96)	5784 (4.42)	<0.001
25–34 years, *n* (%)	1,358,332 (58.03)	3192 (57.4)	2060 (46.91)	65,494 (50)	<0.001
35–39 years, *n* (%)	553,578 (23.65)	1570 (28.23)	1486 (33.84)	44,693 (34.12)	<0.001
40–44 years, *n* (%)	109,299 (4.67)	297 (5.34)	588 (13.39)	13,843 (10.57)	<0.001
≥45 years, *n* (%)	6199 (0.26)	13 (0.23)	83 (1.89)	1166 (0.89)	0.454
Pulmonary hypertension, *n* (%)	112 (0)	<5	<5	8 (0.01)	<0.001
Placenta previa, *n* (%)	11,817 (0.5)	35 (0.63)	27 (0.61)	822 (0.63)	<0.001
Sickle cell disease, *n* (%)	7174 (0.31)	28 (0.5)	16 (0.36)	485 (0.37)	<0.001
Gestational hypertension, *n* (%)	24,462 (1.05)	231 (4.15)	144 (3.28)	3221 (2.46)	<0.001
Mild preeclampsia or unspecified preeclampsia, *n* (%)	21,877 (0.93)	349 (6.28)	198 (4.51)	2431 (1.86)	<0.001
Severe preeclampsia/eclampsia, *n* (%)	10,215 (0.44)	123 (2.21)	74 (1.69)	851 (0.65)	<0.001
Chronic renal disease, *n* (%)	4029 (0.17)	161 (2.9)	62 (1.41)	268 (0.2)	<0.001
Preexisting hypertension, *n* (%)	18,759 (0.8)	268 (4.82)	460 (10.48)	3041 (2.32)	<0.001
Chronic ischemic heart disease, *n* (%)	149 (0.01)	<5	<5	16 (0.01)	<0.001
Congenital heart disease, *n* (%)	1823 (0.08)	12 (0.22)	<5	118 (0.09)	0.001
Systemic lupus erythematosus, *n* (%)	1822 (0.08)	3 (0.05)	<5	81 (0.06)	0.166
Human immunodeficiency virus, *n* (%)	2043 (0.09)	<5	9 (0.2)	104 (0.08)	0.012
Multiple gestation, *n* (%)	50,342 (2.15)	101 (1.82)	98 (2.23)	4475 (3.42)	<0.001
Drug abuse, *n* (%)	4468 (0.19)	13 (0.23)	6 (0.14)	181 (0.14)	<0.001
Alcohol abuse, *n* (%)	764 (0.03)	<5	<5	31 (0.02)	0.035
Tobacco use, *n* (%)	114,001 (4.87)	334 (6.01)	249 (5.67)	7466 (5.7)	<0.001
Cardiac valvular disease, *n* (%)	2219 (0.09)	5 (0.09)	8 (0.18)	160 (0.12)	0.005
Chronic congestive heart failure, *n* (%)	<5	<5	<5	<5	0.981
Asthma, *n* (%)	35,711 (1.53)	67 (1.2)	99 (2.25)	2319 (1.77)	<0.001
Obesity, *n* (%)	38,657 (1.65)	164 (2.95)	474 (10.79)	6896 (5.26)	<0.001
Previous cesarean delivery, *n* (%)	192,441 (8.22)	1033 (18.58)	809 (18.42)	15,144 (11.56)	<0.001
Obstetric Comorbidity Index	978,769 (41.82)	3306 (59.45)	3079 (70.12)	78,960 (60.28)	<0.001

**Table 3:** Impact of diabetes mellitus (DM) types on obstetrical interventions and maternal and neonatal health outcomes.

	Without DM	Type 1 DM	Type 2 DM	Gestational DM
Labor induction. *n* (%)	357,116 (15.26)	1645 (29.58)	1335 (30.4)	29,535 (22.55)
Crude RR (95% CI)		1.94 (1.85–2.03)	1.99 (1.89–2.10)	1.48 (1.46–1.49)
Adjusted RR (95% CI)		1.91 (1.82–2.00)	1.94 (1.84–2.05)	1.45 (1.44–1.47)
Cesarean delivery *n* (%)	505,715 (21.61)	3162 (56.86)	2084 (47.46)	37,828 (28.88)
Crude RR (95% CI)		2.63 (2.54–2.72)	2.20 (2.10–2.29)	1.34 (1.32–1.35)
Adjusted RR (95% CI)		2.34 (2.26–2.43)	1.83 (1.75–1.91)	1.18 (1.17–1.20)
Forceps/vacuum extraction *n* (%)	288,088 (12.31)	650 (11.69)	463 (10.54)	15,523 (11.85)
Crude RR (95% CI)		0.94 (0.88–1.02)	0.86 (0.78–0.94)	0.96 (0.95–0.98)
Adjusted RR (95% CI)		0.96 (0.89–1.04)	0.88 (0.80–0.96)	0.98 (0.96–0.99)
Episiotomy *n* (%)	498,487 (21.3)	690 (12.41)	562 (12.8)	25,113 (19.17)
Crude RR (95% CI)		0.58 (0.54–0.63)	0.60 (0.55–0.65)	0.90 (0.88–0.91)
Adjusted RR (95% CI)		0.61 (0.57–0.66)	0.65 (0.60–0.71)	0.95 (0.94–0.96)
Severe Maternal morbidity *n* (%)	32,744 (1.4)	170 (3.06)	90 (2.05)	2103 (1.61)
Crude RR (95% CI)		2.18 (1.88–2.54)	1.46 (1.19–1.80)	1.15 (1.10–1.20)
Adjusted RR (95% CI)		1.97 (1.70–2.29)	1.25 (1.02–1.54)	1.03 (0.99–1.08)
Prolonged maternal length of stay *n* (%)	245,451 (10.49)	1883 (33.86)	1097 (24.98)	14,662 (11.19)
Crude RR (95% CI)		3.22 (3.09–3.38)	2.38 (2.24–2.53)	1.07 (1.05–1.08)
Adjusted RR (95% CI)		3.09 (2.95–3.23)	2.22 (2.09–2.35)	1.02 (0.99–1.03)
Preterm birth *n* (%)	140,101 (5.99)	1221 (21.96)	660 (15.03)	10,298 (7.86)
Crude RR (95% CI)		3.66 (3.47–3.88)	2.51 (2.33–2.71)	1.31 (1.29–1.34)
Adjusted RR (95% CI)		3.32 (3.14–3.51)	2.15 (1.99–2.32)	1.18 (1.16–1.21)
Fetal overgrowth *n* (%)	28,226 (1.21)	572 (10.29)	322 (7.33)	4419 (3.37)
Crude RR (95% CI)		8.53 (7.85–9.26)	6.08 (5.45–6.79)	2.80 (2.71–2.89)
Adjusted RR (95% CI)		8.05 (7.41–8.75)	5.55 (4.97–6.20)	2.63 (2.55–2.72)

Models adjusted by age and the Obstetric Comorbidity Index. RR Risk Ratio. CI Confidence Interval.

## Supplementary Materials

The following are available online at https://www.mdpi.com/2077-0383/9/2/582/s1, Table S1: Diagnostic Codes Used to Define Maternal Comorbidities, Figure S1: Temporal trends in labor induction; among pregnancies in women with type 1, type 2. and gestational diabetes compared with women without diabetes, Figure S2: Temporal trends in caesarean section among pregnancies in women with type 1, type 2. and gestational diabetes compared with women without diabetes, Figure S3: Temporal trends in prevalence of severe maternal morbidity among pregnancies in women with type 1, type 2 and gestational diabetes compared with women without diabetes, Figure S4: Temporal trends in preterm birth in women with type 1, type 2 and gestational diabetes compared with women without diabetes, Figure S5: Temporal trends in fetal overgrowth among pregnancies in women with type 1, type 2 and gestational diabetes compared with women without diabetes.
