# Supplementary material for: A Population-Based Study of Diabetes during Pregnancy in Spain (2009–2015): Trends in Incidence, Obstetric Interventions, and Pregnancy Outcomes
Source: J Clin Med. 2020 Feb 21;9(2):582. doi: 10.3390/jcm9020582 (PMC7074053; doi:10.3390/jcm9020582)
Supplement: Supplementary file 1 [file jcm-09-00582-s001.zip › jcm-705434-supplementary-figure.docx]

**Supplementary Figure S1.** Temporal trends in labor induction; among pregnancies in women with type 1, type 2. and gestational diabetes compared with women without diabetes.

**Supplementary Figure S2.** Temporal trends in caesarean section among pregnancies in women with type 1, type 2. and gestational diabetes compared with women without diabetes.

**Supplementary Figure S3.** Temporal trends in prevalence of severe maternal morbidity among pregnancies in women with type 1, type 2 and gestational diabetes compared with women without diabetes.

**Supplementary Figure S4.** Temporal trends in preterm birth in women with type 1, type 2 and gestational diabetes compared with women without diabetes.

**Supplementary Figure S5.** Temporal trends in fetal overgrowth among pregnancies in women with type 1, type 2 and gestational diabetes compared with women without diabetes.
