# Supplementary material for: A Population-Based Study of Diabetes during Pregnancy in Spain (2009–2015): Trends in Incidence, Obstetric Interventions, and Pregnancy Outcomes
Source: J Clin Med. 2020 Feb 21;9(2):582. doi: 10.3390/jcm9020582 (PMC7074053; doi:10.3390/jcm9020582)
Supplement: Supplementary file 1 [file jcm-09-00582-s001.zip › jcm-705434-supplementary-table.docx]

Supplementary Table 1. Diagnostic Codes Used to Define Maternal Comorbidities

|  | ICD-9-CM Definition |
| --- | --- |
| Pulmonary hypertension | 416.0x, 416.8x, 416.9x |
| Placenta previa | 641.0x, 641.1x |
| Sickle cell disease | 282.4x, 282.6x |
| Gestational hypertension | 642.3x (without preeclampsia/eclampsia or pre-existing hypertension) |
| Mild preeclampsia or unspecified preeclampsia | 642.4x, 642.7x (without severe preeclampsia/eclampsia) |
| Severe preeclampsia/eclampsia | 642.5x, 642.6x |
| Chronic renal disease | 581.x–583.x, 585.x, 587.x, 588.x, 646.2x |
| Preexisting hypertension | 401.x–405.x, 642.0x–642.2x, 642.7x |
| Chronic ischemic heart disease | 412.x–414.x |
| Congenital heart disease | 745.0x–747.4x, 648.5x |
| Systemic lupus erythematosus | 710.0x |
| Human immunodeficiency virus | 042.x, V08.x |
| Multiple gestation | V27.2–V27.8, 651.x |
| Drug abuse | 304.x, 305.2x–305.9x, 648.3x |
| Alcohol abuse | 291.xx, 303.xx, 305.0x |
| Tobacco use | 305.1.x, 649.0x |
| Cardiac valvular disease | 394.x–397.x, 424.x |
| Chronic congestive heart failure | 428.22, 428.23, 428.32, 428.33, 428.42, 428.43 |
| Asthma | 493.x |
| Obesity | 278.0x, 649.1x ,V85.3, V85.4 |
| Cystic fibrosis | 277.0x |
| Previous cesarean delivery | 654.2x |
